# Shock From Hemorrhagic Ascites in a Non-cirrhotic Patient With Isolated High-Output Right Heart Failure

**DOI:** 10.7759/cureus.88248

**Published:** 2025-07-18

**Authors:** Artemii Lazarev, Jamesina Wong

**Affiliations:** 1 Department of Internal Medicine, Northwest Health, Valparaiso, USA; 2 Department of Medicine, Ross University School of Medicine, Miramar, USA

**Keywords:** abdominal paracentesis, end-stage renal disease (esrd), hemorrhagic ascites, hemorrhagic shock, high-output heart failure

## Abstract

A 49-year-old male patient with multiple comorbidities, including end-stage renal disease (ESRD), diabetes mellitus, obesity, and obstructive sleep apnea, presented to the emergency department with clinical signs of hemorrhagic shock in the setting of volume overload. After initial stabilization, he underwent paracentesis and was diagnosed with hemorrhagic ascites (HA). Post-discharge, the patient was re-admitted multiple times due to refractory HA in the setting of poor volume management. Cirrhosis was ruled out with biopsy, and subsequent workup following his initial presentation eventually revealed high-output right heart failure in the setting of severe pulmonary hypertension (undiagnosed on initial presentation).

Recognizing acute presentations of hemorrhagic ascites secondary to cardiac etiology can prove challenging in a patient with long-standing ESRD in the absence of liver cirrhosis because reported cases in the literature for this specific subset of patient population are seldom. This case will discuss the methodical approach in diagnostic workup taken and emphasize the importance of considering right heart failure as a potential differential, even if a patient had no significant cardiac history on file. More research is needed to further investigate the connection between right heart failure with pulmonary hypertension and hemorrhagic ascites.

## Introduction

Hemorrhagic ascites (HA) is described as ascitic fluid with a red blood cell (RBC) count greater than 10,000/μL [1]. HA is a common complication of late-stage liver disease and affects up to 27% of patients with cirrhosis [1]. In cirrhotic patients, HA may occur spontaneously or because of disseminated malignancy or iatrogenic causes such as traumatic paracentesis and complications secondary to transjugular intrahepatic portosystemic shunt procedures [1,2]. HA in patients without cirrhosis is rare and often enigmatic, although cases of heart failure [3,4], endometriosis [5], hepatic angiosarcoma [6], and splenic lymphoma [7] have been described in the literature as possible etiologies. HA from heart failure can result from intra-abdominal bleeding from small vessels or varices or leakage of blood from liver interstitial spaces [1]; it was described in fluid overload, non-compliant patients.

HA can present with abdominal swelling, tachycardia, tachypnea, hypotension, altered mental status, and low hemoglobin, depending on the severity of the disease course. HA can progress indolently or rapidly. The pathophysiology of indolent HA has been proposed to be a result of an increase in hydrostatic pressure leading to erythrocyte leakage from blood vessels into the peritoneal cavity. Increases in hydrostatic pressure can be attributed to pathological vessel narrowing, intra-abdominal bleeding caused by adjacent organ damage, or portal hypertension [8]. However, this course of HA rarely leads to hemodynamic instability.

Alternatively, rapid and significant loss of intravascular volume may occur as sequelae of ruptured varices and friable hepatocellular cancer tumors or due to traumatic paracentesis [9]. Rapid bleeding into the abdominal cavity may result in hemorrhagic shock. HA is considered a poor prognostic factor, indicating decreased median survival rates in patients with advanced-stage liver disease. Patients with HA are at increased risk for acute kidney injury and intensive care unit (ICU) admissions, and are attributed with higher mortality rates [1,10]. Possible shock secondary to HA were previously described, although the clinical significance of shock in the natural course of hemorrhagic ascites remains unknown [5,11,12]. We present a case of hemorrhagic shock secondary to cardiac cause of HA in a man with no medical history of cirrhosis. After the case presentation, we will discuss further diagnostic workup and our considerations regarding the etiology of his HA.

## Case presentation

A 49-year-old East Asian man presented to the emergency department with acute signs and symptoms of suspected hemorrhagic shock. Due to the patient’s altered mental state, a clear medical history could not be obtained. Upon initial chart review, the patient’s past medical history was significant for end-stage renal disease (ESRD), obesity, obstructive sleep apnea, asthma, history of remote alcohol abuse, and worsening refractory ascites. Other diagnoses included uncontrolled type 2 diabetes mellitus, chronic diarrhea, hypertension, and hyperlipidemia. The patient arrived with documents from another institution, from which he was discharged three days prior. At that institution, he was diagnosed with *Clostridioides difficile *(*C. difficile*) colitis via a positive *C. difficile* toxin polymerase chain reaction (PCR) stool test. He was instructed to take oral antibiotics at home, and the patient reluctantly admitted that he did not finish his course. He noted that he continued to experience intractable diarrhea, which caused him to miss his hemodialysis sessions.

On examination, vitals were notable for altered mental status, blood pressure of 88/62 mmHg, and hypothermia at 35.5°C. His body mass index (BMI) was 29 kg/m^2^, which showed a gradual decrease from previous hospitalizations. Physical examination revealed abdominal distention with tenderness in all four quadrants. Shifting dullness was positive, but no hepatosplenomegaly was detected. Moderate bilateral edema of the lower limbs was also noted. At the time of presentation, given his instability (hypotension and altered mentation), the initial differential diagnosis included septic shock, uremia, and untreated or fulminant *C. difficile* infection. Fluid resuscitation was withheld due to concerns for fluid overload through third spacing, given his leg swelling, ascites, and history of ESRD and missed hemodialysis sessions. The patient progressively became more somnolent as initial bloodwork was pending. A central line was placed, and continuous norepinephrine infusion was initiated to correct hypotension.

When the initial results for his complete blood count with differentials returned, it revealed an alarmingly low hemoglobin level of 5.5 g/dL, along with decreased RBC and hematocrit, which indicates severe anemia lower than the general transfusion threshold of 7 g/dL, and the other causes of his clinical picture became less likely. This was a notable decrease from 8.3 g/dL three days prior. Hemorrhagic shock was subsequently added to the initial differential diagnoses. The patient’s initial laboratory results are shown in Table 1 and Table 2. Elevated blood urea nitrogen (BUN) and creatinine reflect the patient’s ESRD, and rising anion gap and abnormal CO2 raise suspicion for anion-gap metabolic acidosis from uremia; increased potassium is not uncommon in ESRD patients. Low calcium might reflect chronic kidney disease-mineral bone disorder.

**Table 1:** Initial admission complete blood count with differentials RBC: red blood cell, MCV: mean corpuscular volume, MCH: mean corpuscular hemoglobin, MCHC: mean corpuscular hemoglobin concentration, RDW: red cell distribution width, MPV: mean platelet volume, WBC: white blood cell

Test	Value	Reference range
RBC	1.89 × 10^6^/mcL	4.34-5.60 × 10^6^/mcL
Hemoglobin	5.5 g/dL	12-17 g/dL
Hematocrit	16.9%	38.6%-49.2%
MCV	89.3 fL	80-100 fL
MCH	29.2 pg	26-34 pg
MCHC	32.7 g/dL	32.5-35.8 g/dL
Platelet count	170 × 10^3^/mcL	150-450 × 10^3^/mcL
RDW	15.9%	11.5%-15.9%
MPV	7.4 fL	6.8-10.2 fL
WBC	5.60 × 10^3^/mcL	4-11 × 10^3^/mcL
Neutrophils	66%	43%-82.3%
Lymphocytes	11%	14.5%-45.2%
Monocytes	15%	4.3%-13.3%
Eosinophils	6%	0.1%-6.8%
Basophils	2%	0%-2%
Neutrophils (absolute)	3.70 × 10^3^/mcL	1.7-7.70 × 10^3^/mcL
Lymphocytes (absolute)	0.62 × 10^3^/mcL	0.60-3.40 × 10^3^/mcL
Monocytes (absolute)	0.84 × 10^3^/mcL	0.30-1.00 × 10^3^/mcL
Eosinophils (absolute)	0.34 × 10^3^/mcL	0.00-0.50 × 10^3^/mcL
Basophils (absolute)	0.11 × 10^3^/mcL	0.00-0.20 × 10^3^/mcL

**Table 2:** Initial and repeat comprehensive metabolic panel BUN: blood urea nitrogen, GFR: glomerular filtration rate, SGOT: serum glutamic-oxaloacetic transaminase, AST: aspartate aminotransferase, SGPT: serum glutamic-pyruvic transaminase, ALT: alanine aminotransferase

Test	Value on admission	Value 3 hours after admission	Reference range
Sodium	143 mEq/L	138 mEq/L	133-144 mEq/L
Potassium	4.1 mEq/L	5.9 mEq/L	3.5-5.2 mEq/L
Chloride	103 mEq/L	101 mEq/L	98-107 mEq/L
CO2 (venous)	25.9 mEq/L	20.7 mEq/L	21-31 mEq/L
Anion gap	14.1 mEq/L	16.3 mEq/L	4-11 mEq/L
BUN	47 mg/dL	92 mg/dL	7-25 mg/dL
Creatinine (blood)	8.05 mg/dL	14.83 mg/dL	0.70-1.30 mg/dL
GFR (estimated)	7	3	≥60
BUN/creatinine ratio	6	6	5-19
Glucose	99 mg/dL	111 mg/dL	70-99 mg/dL
Calcium	8.5 mg/dL	7.4 mg/dL	8.6-10.3 mg/dL
Total bilirubin	0.4 mg/dL	0.4 mg/dL	0.3-1.0 mg/dL
SGOT (AST)	44 U/L	45 U/L	13-33 U/L
SGPT (ALT)	35 U/L	32 U/L	7-51 U/L
Alkaline phosphatase	78 U/L	73 U/L	34-104 U/L
Total protein	6.4 g/dL	6 g/dL	6.4-8.9 g/dL
Albumin	3.8 g/dL	3.6 g/dL	3.5-5.7 g/dL
A/G ratio	1.5	1.5	0.8-1.8

The patient was subsequently transferred to the medical ICU for further management with urgent hemodialysis and transfusion. Two units of packed red blood cells and calcium gluconate were administered to correct for low hemoglobin levels and hyperkalemia, respectively. Intravenous antibiotics (metronidazole and cefepime) were given prophylactically for concerns of sepsis and possible intra-abdominal infections. Shortly after, the patient had two episodes of coffee ground emesis and was started on an intravenous proton pump inhibitor.

Following hemodialysis and transfusion, the patient’s blood pressure improved, and he was gradually weaned off vasopressors. His mentation improved significantly, and he became coherent enough to provide a clear medical history. Further diagnostic imaging was performed with chest X-ray and computed tomography (CT), which showed no evidence of pulmonary infection or edema and the absence of acute intracranial processes, respectively.

**Figure 1:**
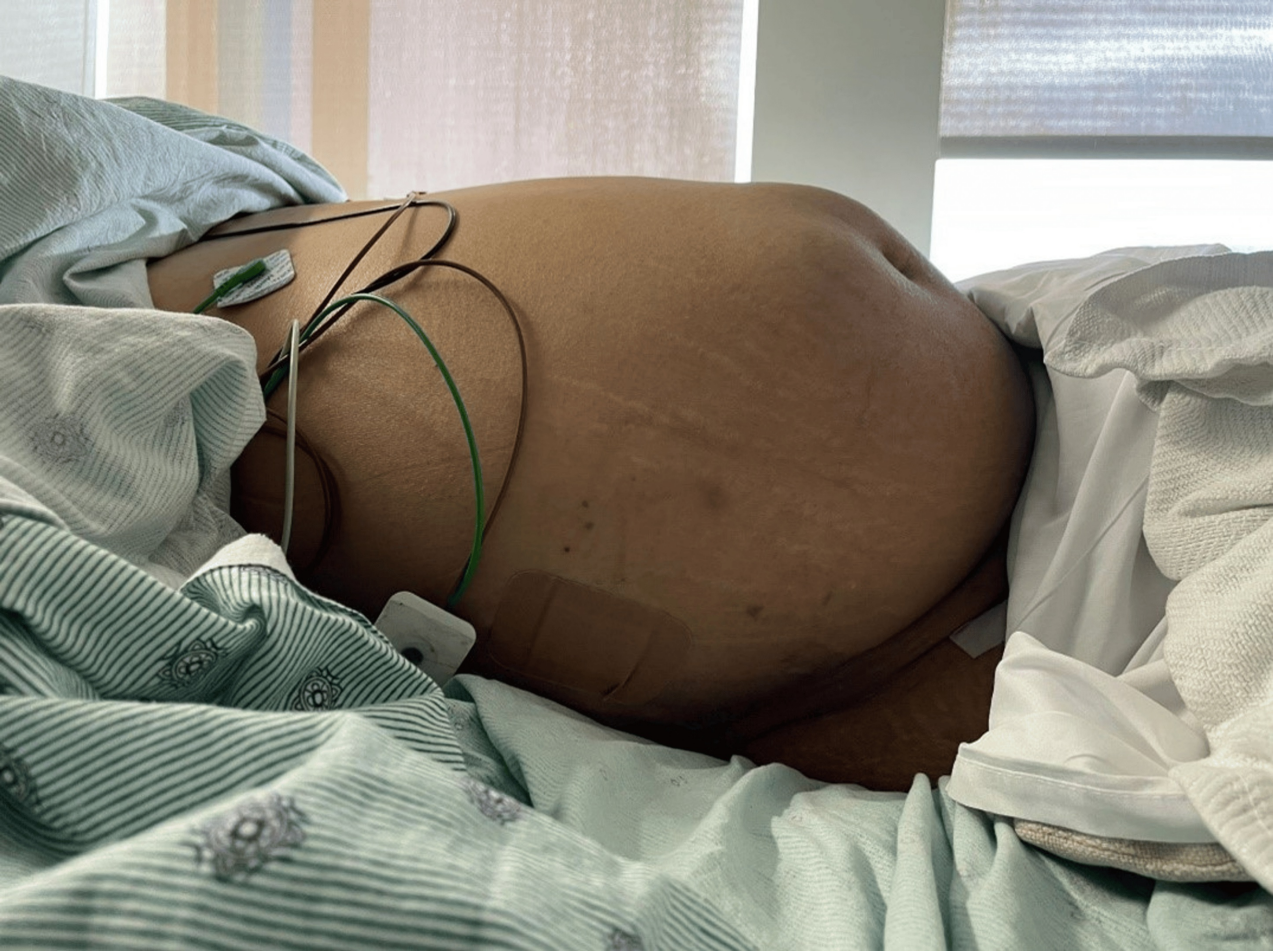
Patient’s abdomen, which was abnormally distended despite recent therapeutic paracentesis three days prior at a different hospital This may indicate that the patient has re-accumulated ascitic fluid in the abdomen. An evaluation for ascites should be performed in that situation.

The patient continued to complain about significant abdominal discomfort despite noting that he had undergone therapeutic paracentesis at another facility (Figure 1). The patient disclosed that he had two separate paracentesis procedures (attempted and aborted due to technical reasons and performed) four and three days ago. He endorsed that there was no fluid drawn during the first attempt, and his procedure was repeated the following day prior to his discharge. The second attempt was successful, and straw-colored fluid was obtained from his abdomen. As per the patient, there were no complications, and he tolerated the procedure well.

To ameliorate the patient’s current abdominal discomfort secondary to recurrent intra-abdominal ascites, therapeutic paracentesis was performed. Approximately 60 mL of bloody peritoneal ascitic fluid were collected and sent for laboratory analysis. An additional 5 L of blood-tinged ascitic fluid was drained.

**Figure 2:**
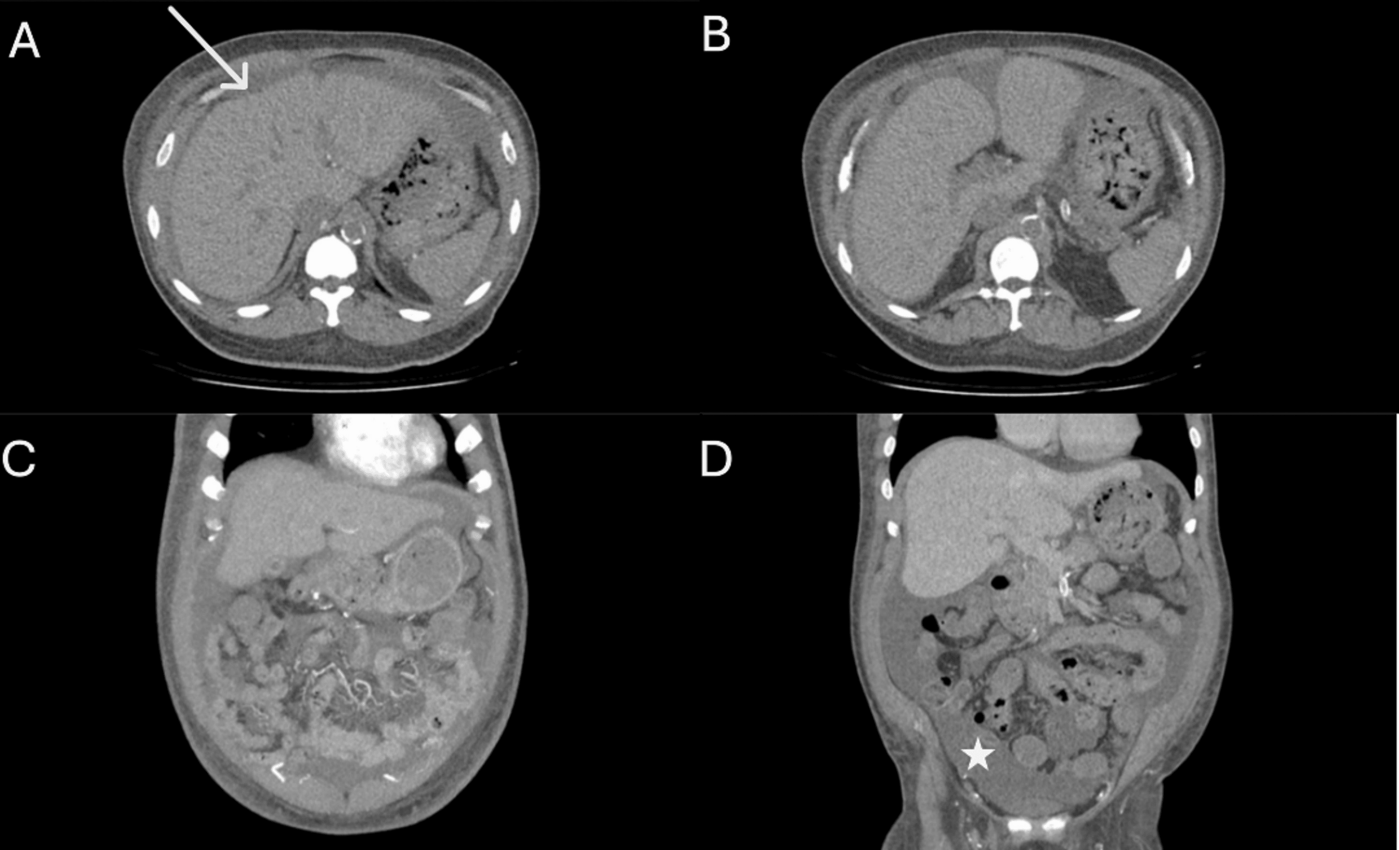
Computed tomography of the abdomen Transverse (A and B) and coronal (C and D) abdominal computed tomography axes demonstrating mild hepatomegaly and mild nodular liver contour (arrow) and ascites (asterisk). The liver findings, along with the patient’s clinical presentation, subsequently led to a liver biopsy, which did not reveal cirrhosis.

Fluid analysis yielded a significantly increased RBC count close to 600,000 RBC/mm^3^, as shown in Table 3. In order to detect the cause of his ascites, the serum ascites albumin gradient (SAAG) was calculated as 1.4 g/dL (≥1.1 g/dL, indicative of portal hypertension), and in the context of total protein of 3.8 g/dL, a cardiac etiology of ascites was considered, given the markedly elevated B-type natriuretic peptide (BNP) level of over 2,500. Repeat hemoglobin after the procedure showed a decrease from 9 to 7.5 g/dL, and the concern for bleeding was raised. Computed tomography angiography (CTA) of the abdomen and pelvis was performed in order to detect hemorrhage and revealed no active bleeds. CTA imaging showed slight liver enlargement with mild nodular contour, trace right-sided pleural effusion with adjacent compressive atelectasis, and a moderate amount of intra-abdominal ascites (Figure 2).

**Table 3:** Analysis of ascitic fluid during current admission RBC: red blood cell, WBC: white blood cell, AFB: acid-fast bacteria

Test	Value/result
Color	Red
Clarity	Bloody
Volume	8
Fluid RBC count	595,556/mm^3^
Fluid WBC	500/mm^3^
Total count fluid differential	100
Fluid neutrophils	13%
Fluid lymphocytes	10%
Fluid monocytes/macrophages	34%
Fluid eosinophils	29%
Fluid basophils	-
Fluid lining cells	14%
Fluid plasma cells	-
Fluid endothelial cells	-
Fluid other cells	-
AFB culture	Negative
Body fluid gram stain culture	Negative
Albumin peritoneal fluid	2.4 g/dL
Glucose	83 mg/dL
Total protein	3.8 g/dL

This raised concerns for refractory ascites as a result of potential liver cirrhosis. When questioned, the patient admitted to years of heavy alcohol use since late adolescence but claimed 10 years of sobriety. Additional infectious and immunological studies were ordered to further evaluate possible causes of hepatic cirrhosis (Table 4 and Table 5). His overall condition improved, and he was discharged with the plan for close follow-up with a gastroenterologist and his primary care physician.

**Table 4:** Additional infectious testing HIV: human immunodeficiency virus

Test	Result
Hepatitis B surface antigen	Non-reactive
Hepatitis B core antibody, total	Non-reactive
Hepatitis B core antibody, IgM, total	Non-reactive
Hepatitis B surface antibody, total	Reactive
Hepatitis A antibody, IgM	Non-reactive
Hepatitis C antibody, IgG screen	Non-reactive
Hepatitis B surface antigen	Non-reactive
HIV Ag/Ab combo, qualitative	Non-reactive

**Table 5:** Autoimmune panel for hepatitis DS DNA: double-stranded DNA, ANA: antinuclear antibody, ANCA: antineutrophil cytoplasmic antibody, SSA: Sjögren's syndrome A, SSB: Sjögren's syndrome B

Test	Value	Reference range
DS DNA antibodies	1 IU/mL	≤4 IU/mL
Mitochondrial antibodies	<1:20	<1:20
ANA titer	<1:40	<1:40
ANCA screen	Negative	-
Myeloperoxidase antibodies, IgG	<1.0	<1.0
ANCA, proteinase-2 antibodies	<1.0	<1.0
Smooth muscle antibodies	<1:20	<1:20
Rheumatoid factor	<10.0 IU/mL	<14.0 IU/mL
Scleroderma (Scl-70) antibodies	<0.2	<1.0
SSA antibodies	<0.2	<1.0
SSB antibodies	<0.2	<1.0 AI

Subsequently, after discharge, the patient was re-admitted on seven separate occasions due to refractory ascites and continuously missed hemodialysis, which required therapeutic paracentesis procedures and urgent volume management. Out of seven fluid analyses, six were indicative of hemorrhagic ascites with elevated RBC counts of up to 712,000 RBC/mm^3^. In all seven instances, the calculated SAAG values were greater than 1.1 g/dL, and ascitic total protein values were elevated over 2.5 g/dL. Acid-fast bacilli culture and gram stain culture were negative for growth. Alpha-fetoprotein tumor marker and carcinoembryonic antigen were insignificant, and cytology showed fibrin with reactive mesothelial cells and macrophages, but no malignant cells. These findings were consistent with acute obstructive hepatic causes or cardiac etiologies of ascites. Abdominal ultrasound revealed a patent main, left, and right portal vein with normal hepatopetal flow. There was no evidence of intrahepatic ductal dilations.

Following initial hospitalization, he underwent interventional radiology-assisted transjugular liver biopsy to assess for cirrhosis and hepatic portal hypertension as a possible cause of hepatic ascites. Transjugular liver biopsy was negative for cirrhosis, and measurements taken included the following: right atrial pressure (18 mmHg), inferior vena cava pressure (21 mmHg), free hepatic venous pressure (reflecting systemic venous pressure) (23 mmHg), and wedged hepatic venous pressure (reflecting portal venous system pressure) (26 mmHg). His hepatic venous pressure gradient was, thus, 3 mmHg, which is normal, ruling out clinically significant portal hypertension.

**Figure 3:**
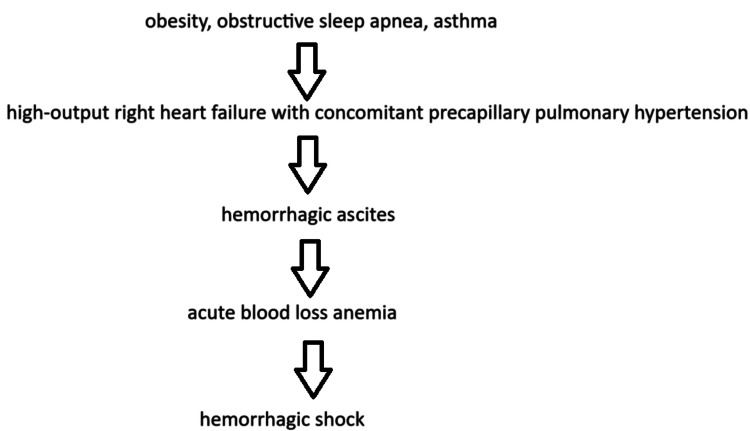
Graphical summary of pathophysiological connections between the patient’s diagnoses

Due to consistently elevated BNP levels that varied from >2,500 to 14,000 pg/mL, a transthoracic echocardiography (TTE) was obtained and showed a grossly normal left ventricular cavity size and mildly increased wall thickness, with a left ventricular ejection fraction of approximately 55%-60% and normal systolic function. The right atrium and ventricle were dilated in size. The mean pulmonary artery pressure was estimated to be increased at 54 mmHg. He had echocardiographic changes consistent with both volume and pressure overload. The mitral valve was mildly calcified and thickened, and there was evidence of moderate tricuspid valve regurgitation. Furthermore, a small extra-cardiac pericardial effusion was identified circumferentially to the heart. The patient subsequently underwent right heart catheterization (RHC), which is the gold standard to aid in diagnosing pulmonary hypertension [13]. This procedure showed mildly increased right ventricle size, normal right ventricle function, pulmonary artery systolic pressure of 80 mmHg, pulmonary capillary wedge pressure of 12 mmHg, elevated mean pulmonary artery pressure of 32 mmHg, cardiac output of 8.3 L/minute, and cardiac index of 4.2 L/minute/m². Pulmonary vascular resistance was measured at 4.8 Woods units. The RHC data were suggestive of precapillary pulmonary hypertension (elevated mean pulmonary artery pressure in the setting of normal pulmonary artery wedge pressure and elevated pulmonary vascular resistance). He also had evidence of volume and pressure overload, and the systolic function of the right and left heart was normal. Given the elevated cardiac index in the setting of clinical heart failure, we could describe his pattern of cardiovascular disease as high-output right heart failure with concomitant precapillary pulmonary hypertension. Figure 3 provides an overall graphical summary of the pathophysiological connections between the nosological entities in our case.

## Discussion

We describe the case of an unstable patient showing evidence of hemorrhagic shock in the setting of HA. Despite rapid improvement with appropriate resuscitative measures, including blood transfusion, further diagnostic search was enigmatic. Fluid analysis revealed an elevated SAAG score and total protein levels. The patient underwent a liver biopsy, which showed no cirrhosis or significant portal hypertension. Infectious and autoimmune workups were negative as well. Cardiac workup showed evidence of right heart failure, precapillary pulmonary hypertension, and an elevated cardiac index. Despite meeting criteria for normal systolic heart function, there was compelling evidence of right pressure and volume overload, making cardiac ascites a more likely etiology.

Apart from heart failure and cirrhosis, HA might be caused by several other causes: endometriosis, sarcoidosis, tuberculosis, ruptured ovarian cyst, and malignancies, such as hepatocellular carcinoma and splenic lymphomas. It is important to rule out malignancy and infection in every patient with HA. In our patient, peritoneal fluid examinations for acid-fast bacilli, malignant cytology, and bacterial cultures were negative. Alpha-fetoprotein was negative, making malignant and infectious causes of HA less likely in our case, and abdominal ultrasound showed potent hepatic vasculature, ruling out Budd-Chiari syndrome.

Hemorrhagic ascites secondary to cardiac etiology has been previously described in other cases. In a 1998 study by Goel et al., a 65-year-old male patient with nephropathy, left ventricular dysfunction, biventricular heart failure, and medical non-compliance presented with stable vitals, excluding minimal tachycardia of 104/minute. Workup revealed a SAAG of 1.2 g/dL and an RBC count of 22,000/mm^3^. Malignancy and infectious workup came back negative. This patient was started on medical therapy for heart failure with improvement in clinical condition, but was eventually re-admitted for HA with an RBC ascitic fluid count of 560/mm^3^ [4]. Another study by Abbarh et al. presented a 64-year-old woman with a history of chronic kidney disease and heart failure with preserved ejection fraction who presented with HA secondary to heart failure [3] and recurrent admissions due to medical non-compliance. The patient had an SAAG of 14.8 g/L, RBC ascitic fluid count of 75,000 RBC/mm^3^, and total ascitic protein of 28.7 g/dL. Transthoracic echocardiography of this patient revealed similar findings to our case of dilatation of both right atria and ventricle, severe tricuspid regurgitation, and elevated pulmonary artery pressure of 71 mmHg. However, in Abbarh et al.’s study, their patient’s hemoglobin was stable at 10 g/dL as they received aggressive diuresis and was eventually discharged on a higher dose of diuretic after optimizing heart failure therapy. Additionally, compared to the aforementioned works, our patient initially presented in a much more critical condition. He was obtunded, hypothermic, and hypotensive, with severe anemia requiring transfusion. The RBC ascitic fluid count was 595,000 RBC/mm^3^, which was much higher than that described by Goel et al. [4] and Abbarh et al. [3].

Another distinctive feature of our case report is the availability of RHC data. Our patient had high-output right heart failure with concomitant precapillary pulmonary hypertension. High-output heart failure is relatively rare and is commonly associated with obesity and lung disease [14], which our patient had. The connection between hemorrhagic ascites and high-output heart failure needs to be further assessed in future studies.

The possible mechanisms of HA in the setting of heart failure include intra-abdominal bleeding from small vessels or varices or leakage of blood from liver interstitial spaces [1,15]. As previously explained, iatrogenic complications such as trauma from previously performed paracentesis can result in rapid and life-threatening HA. However, serial presentations with serosanguinous exudates denote a more indolent course of HA, which rarely results in hemodynamic instability [8]. We would argue that these recurrent presentations of hypervolemia were the consequence of non-compliance with heart failure medication and missed hemodialysis sessions. Similar to the cases reported by Goel et al. [4] and Abbarh et al. [3], the patients were non-compliant with their heart failure therapy. Thus, we can speculate that in HA cases secondary to heart failure, volume management is of particular importance. We would also like to emphasize that recurrence of HA in patients with heart failure is common, as in the cases of Abbarh et al. [3] and Goel et al. [4], and in all three cases, recurrent presentations are milder. Notably, to our knowledge, our case was the first to describe a presentation severe enough to require vasopressors and ICU stay, as well as packed RBC transfusion.

The management of refractory hemorrhagic ascites depends largely on the underlying cause [16]. Currently, there are no clear guidelines for managing hemorrhagic ascites related to cardiogenic causes of congestion and volume overload. In patients with HA from heart failure, in addition to meticulous control of volume status with appropriate diuretic/other volume management therapy, including dialysis, potentially decreasing rates of HA re-occurrence, therapeutic paracentesis can be done as needed. Clearance of recurrent ascites in cirrhotic patients is achieved by repeat paracentesis and aggressive diuresis as a form of decongestive therapy, and transjugular intrahepatic portosystemic shunts appear to be the mainstay treatment to provide patients with symptomatic relief by minimizing the frequency and severity; however, it is not curative [16]. A meta-analysis carried out by Salerno et al. provided promising evidence of survival rates as high as 80% at three years in patients with normal liver function and sodium levels [17].

## Conclusions

Recognizing acute presentations of hemorrhagic ascites secondary to cardiac etiology can prove challenging in a patient with long-standing ESRD in the absence of liver cirrhosis, because reported cases in the literature for non-cirrhotic patients are seldom. We describe the case of hemorrhagic shock in a patient who initially presented without a significant cardiac history. He was found to have hemorrhagic ascites, which then re-occurred, and a lot of effort was made to find the explanation for that. Diagnostic workup eventually revealed cardiac ascites and high-output right heart failure with precapillary pulmonary hypertension, which led to recurrent cardiac ascites. Based on our and other cases, we hypothesize that cardiac ascites might recur in patients who are non-compliant with volume management. Further studies are needed to assess the correlation between heart failure patterns (case series in heart failure patients, e.g., high-output and right ventricular) and the resulting presentations of hemorrhagic ascites (recurrence versus remission with appropriate management) in non-cirrhotic patients. Long-term therapeutic strategies are still unclear, although volume management seems to be important; cohort studies might clarify that. Finally, high-output heart failure must be considered when evaluating and treating patients with hemorrhagic ascites.
